# Discriminating the Drivers of Edge Effects on Nest Predation: Forest Edges Reduce Capture Rates of Ship Rats (*Rattus rattus*), a Globally Invasive Nest Predator, by Altering Vegetation Structure

**DOI:** 10.1371/journal.pone.0113098

**Published:** 2014-11-20

**Authors:** Jay Ruffell, Raphael K. Didham, Paul Barrett, Nic Gorman, Rhonda Pike, Andrée Hickey-Elliott, Karin Sievwright, Doug P. Armstrong

**Affiliations:** 1 School of Animal Biology, The University of Western Australia, Perth, Australia; 2 Centre for Environment and Life Sciences, the Commonwealth Scientific and Industrial Research Organisation, Perth, Australia; 3 Wildlife Ecology Group, Te Kura Mātauranga o ngā Taonga ā Papatuanuku, Massey University, Palmerston North, New Zealand; Dauphin Island Sea Lab, United States of America

## Abstract

Forest edges can strongly affect avian nest success by altering nest predation rates, but this relationship is inconsistent and context dependent. There is a need for researchers to improve the predictability of edge effects on nest predation rates by examining the mechanisms driving their occurrence and variability. In this study, we examined how the capture rates of ship rats, an invasive nest predator responsible for avian declines globally, varied with distance from the forest edge within forest fragments in a pastoral landscape in New Zealand. We hypothesised that forest edges would affect capture rates by altering vegetation structure within fragments, and that the strength of edge effects would depend on whether fragments were grazed by livestock. We measured vegetation structure and rat capture rates at 488 locations ranging from 0–212 m from the forest edge in 15 forest fragments, seven of which were grazed. Contrary to the vast majority of previous studies of edge effects on nest predation, ship rat capture rates increased with increasing distance from the forest edge. For grazed fragments, capture rates were estimated to be 78% lower at the forest edge than 118 m into the forest interior (the farthest distance for grazed fragments). This relationship was similar for ungrazed fragments, with capture rates estimated to be 51% lower at the forest edge than 118 m into the forest interior. A subsequent path analysis suggested that these ‘reverse’ edge effects were largely or entirely mediated by changes in vegetation structure, implying that edge effects on ship rats can be predicted from the response of vegetation structure to forest edges. We suggest the occurrence, strength, and direction of edge effects on nest predation rates may depend on edge-driven changes in local habitat when the dominant predator is primarily restricted to forest patches.

## Introduction

The majority of the world's forests are fragmented by human activities to some extent [1], and the species that occur within them are vulnerable to the impacts of forest fragmentation. Moreover, the extent of forest fragmentation is likely to increase dramatically into the foreseeable future as the human population and its resource requirements grow [2], [3]. Understanding the impacts of forest fragmentation on ecological systems is therefore fundamentally important for effective conservation management in many regions, and has become a major focus for conservation research in recent decades [4].

One potential impact of forest fragmentation that has received considerable attention is the effect of forest edges on predation rates of forest birds' nests [5], [6]. Nest predation is the major cause of nest failure in birds, and is believed to strongly influence the population dynamics of many species [7], [8]. Causes of variability in nest predation rates are therefore of substantial interest to ecologists and conservation managers [6], and there are now many hundreds of studies describing the relationship between nest predation rates and distance from the forest edge.

As a result of this research, forest edges are widely believed to reduce nest success by increasing rates of nest predation [9]. However, although several reviews of the topic have shown that predation rates can indeed be greater at forest edges [10]–[12], there is a growing body of evidence which suggests that these effects are inconsistent and context dependent [9], [10], [13]–[16]. This variability presents a significant issue for predicting and managing the effects of forest fragmentation on birds. The challenge for researchers is to understand the factors that give rise to edge effects, as well as those that moderate their influence, so that the impacts of fragmentation can be predicted and managed effectively [4], [5], [17]. For this reason, there is a need for researchers to move beyond simply describing edge effects on nest predation rates to identifying the mechanisms underlying their occurrence and strength [5], [18], [19]. In particular, because edge effects on nest predation are likely to depend to a large degree on the response of predators to fragmentation-induced changes in habitat, a primary focus of research should be the relationships between forest edges, habitat characteristics, and the abundance or activity of dominant nest predators [6], [15], [20]. This point has largely been overlooked, but might well be the key to understanding the observed variability in edge effects on nest predation [6].

Additionally, because variability in edge effects is likely to be due, at least in part, to differences among predators [6],[12],[13],[16],[21], there is an urgent need for studies of edge effects on nest predation in a more representative range of regions [11]. At present, the vast majority of studies have been conducted in North America or Europe, with only a handful in the southern hemisphere [11], [13]. Overcoming this geographical bias will be important both for region-specific management and for providing insights into the generality of existing theory which has been derived primarily from North American and European systems.

The ship rat (*Rattus rattus*; also known as the black rat) is a globally invasive nest predator which has been widely implicated in the declines of birds and other taxa in many regions, particularly where prey species are naive to mammalian predators [22], [23]–[30]. In New Zealand, for example, ship rats are thought to be the primary predator of the eggs and chicks of forest birds [31]. However, despite their major impact on the viability of bird populations in many systems, we know of only two studies that have examined the effects of forest edges on ship rats. Delgado et al. [32] examined the effects of forest edges on seed predation rates by ship rats in the Canary Islands. They found that predation rates were higher at the forest edge than in the forest interior, and hypothesised that higher vegetation density at the forest edge may have been responsible. In contrast, Christie et al. [33] found that ship rat capture rates in New Zealand forests were lower at the forest edge, although they did not discuss potential reasons for this finding.

In this study, we examined the effects of forest edges on ship rat capture rates (which we used as a proxy for nest predation risk) in forest fragments in a pastoral landscape in New Zealand. We took a mechanistic approach, examining how local habitat characteristics might give rise to edge effects as well as causing variability in those effects. Based on previous findings that ship rat encounter rates correlate with vegetation structure [34], [35] and that vegetation structure is often affected by forest edges [5], we hypothesised that effects of forest edges on rat capture rates would operate via changes in vegetation structure within forest patches. Moreover, because vegetation structure can also be strongly affected by livestock grazing [36], [37], we additionally hypothesised that the effects of forest edges on rat capture rates would vary depending on whether forest fragments were grazed by livestock. Such an interaction could arise, for example, if grazing overwhelmed any effects of edge distance on vegetation structure or if grazing itself was concentrated at the forest edge.

Our specific aims were to (1) examine whether ship rat capture rates vary with distance from edge in forest fragments in New Zealand; (2) examine if livestock grazing causes variability in the strength of this relationship; and (3) determine the mechanisms underlying both the occurrence of edge effects and variability in their strength, by testing the hypothesis that edge effects on rat capture rates are mediated by changes in vegetation structure.

## Methods

### Study design and data collection

The study took place near the town of Benneydale (75° 220′E, 38° 320′S), in the Waikato region of central North Island. The landscape consists primarily of remnants of mature native podocarp-broadleaf forest dominated by tawa (*Beilschmiedia tawa*) in a matrix of pastoral farming. Native forest cover is approximately 23% of the landscape, as measured in ArcGIS (version 10.0) from the New Zealand Land Cover Database in a 10 km radius from the centre of the study area. Remnants in the area contain most of the common native and exotic birds found in forests throughout mainland New Zealand, and many of these native species are believed to be affected by nest predation from ship rats [22]. Most notably, the viability of a North Island robin (*Petroica longipes*) population distributed among these remnants has been shown to depend on a low abundance of ship rats [38].

Rats were sampled in 13 forest patches, ranging from 2–19 ha. Six of these were fenced to exclude livestock, five were grazed, and a further two contained a fenced and an unfenced section. The fenced and unfenced sections of these partly fenced patches were treated as separate patches for our analyses, and hereafter we refer to a sample size of 15 patches. There was no targeted rat control in any of the patches at the time the study began. All patches were on private land, with land access permissions obtained through the Tiroa E & Te Hape B Trusts (http://www.tiroatehape.maori.nz). Specific site locations are provided as GPS coordinates in Supporting Information.

Rats were captured during a kill trapping program which was part of a wider study on the effects of ship rats on North Island robins. Victor snap traps, placed within handmade corflute tunnels and pegged to the ground, were laid out on a 50 m grid throughout the entire area of each patch. These were left in place for 4–5 months prior to trapping to reduce rat neophobia, then baited with peanut butter, set, and checked the following day to see if they caught a rat. Individual patches were trapped in August of either 2008 or 2009, with no replication across years. Trapping was conducted on the same night for all patches within each year. The Massey University Animal Ethics Committee determined that trapping did not require ethics approval, but the traps used pass the humaneness standard for vertebrate control set by New Zealand's National Animal Welfare Advisory Committee [39]. The study did not involve any protected or endangered species.

In total, we deployed 488 traps across our 15 patches, with the number of traps per patch ranging from 5–76 (mean  = 35). The location of each trap was recorded with a handheld GPS. We used Google Earth's drawing tools to create polygons that mapped the boundary of each forest patch, then used ArcGIS to measure the distance from each trap to the nearest polygon boundary. We estimate measurement error for patch boundaries to be ∼ +/−10 m, a similar scale to GPS measurement error. Estimated distance of traps from the forest edge ranged from 0–212 m. Patch sizes were similar between grazed and ungrazed patches (Table S1).

We used the binomial data on whether a trap caught a rat over a single night as the response variable in our statistical models (see below). We chose this response variable because of its similarity to the ‘rat tracking rate’ (RTR) index, which measures the proportion of footprint tracking tunnels tracked by rats (or equivalently, the probability that an individual tunnel will be tracked) over a single night, for tunnels spaced at 50 m and baited with peanut butter. The RTR index is the standard protocol used as a proxy for rat abundance in New Zealand [40], and has been shown to relate to predation risk for several bird species [41]–[43]. Moreover, it may be a better measure of nest predation rates than artificial nests [34]. As a result, we believe that our response variable was likely to reflect the risk of nest predation from ship rats. The use of snap traps within our tunnels allowed us to identify rats as ship rats (as opposed to Norway rats, *Rattus norvegicus*, which are also occasionally caught in mainland New Zealand forests [31]).

To examine whether effects of edge distance on rat capture rates operated via changes in vegetation structure, we took a number of measurements to quantify the structure of the vegetation in a 15 m radius around each trap: (1) understorey density, scored as either 0 (‘sparse’), 1 (‘average’), or 2 (‘dense’); (2) percent cover of dead tree fern fronds on the ground; (3) percent cover of living vegetation <1.5 m above ground; (4) percent canopy cover; (5) average canopy height, scored as either 0–5 m, 5–15 m, or >15 m; and (6) presence of vines (supplejack, *Ripogonum scandens*). We selected this set of variables because we believed that together they captured the variation in vegetation structure that we observed among our trap sites. Variables 1–5, which were difficult to measure objectively, were measured by two observers and averaged. Vegetation structure data, and all other data used in this study, are provided in Table S4.

### Statistical analyses

#### Net effects of distance from edge and livestock grazing on rat capture probability

Initially, we used binomial generalised linear mixed models (‘GLMMs’) to examine the net effects of distance from forest edge, livestock grazing, and their interaction on the probability of rat capture (i.e. without examining the hypothesised pathways via altered vegetation structure). GLMMs were fitted using the ‘lme4’ package [44] in R version 2.13.1 [45]. We used binomial GLMMs because trapping outcome was a binary variable, and because of the hierarchical structure of our data (traps nested within patches). Patch was included as a random factor in these models to account for this nesting.

We developed five candidate models that included all combinations of distance from edge, livestock grazing, and their interaction as predictors of rat capture probability, including an intercept-only null model, then used AIC to estimate which of these models best described variation in rat capture probability. Where there were multiple models that were reasonably well supported by the data (i.e. a ΔAIC of <4 [46]), we used the ‘model.avg’ function in the R package ‘MuMIn’ [47] to average parameter estimates across these models. This approach reduces bias in parameter estimates when multiple models are similarly likely [46].

#### Testing the role of vegetation structure in edge effects and livestock grazing effects

We used path analysis to test our hypothesis that distance from edge, livestock grazing, and their interaction affected rat capture probability via changes in vegetation structure. Path analysis allowed us to test support for this hypothesised causal structure, and to estimate the strength of each relationship while accounting for all of the relationships implied by the causal structure [48]. We used generalised multilevel confirmatory path analysis [49], which allowed us to account for the nesting of traps within forest patches and binomial rat capture data [49].

Generalised multilevel confirmatory path analysis does not allow for reciprocal effects among variables [49]. However, most of our six measured vegetation variables were correlated with one another, and we could not rule out the possibility that they were reciprocally related. For example, a high cover of dead tree fern fronds was likely to reduce the recruitment of seedlings into the understorey, while at the same time a dense understorey was likely to suppress the growth of tree ferns. To avoid the possibility of reciprocal relationships, we took the conservative approach of including only two vegetation variables in our path models: understorey density and presence of vines. These two variables were not correlated with each other but together were correlated with all other vegetation variables.

However, this conservative approach could potentially underestimate the extent to which vegetation structure mediated the effects of forest edges and livestock grazing on rat capture rates, because any variation in vegetation structure that was not captured by understorey density or the presence of vines could be attributed to paths not mediated by these variables. We therefore conducted a sensitivity analysis to examine whether the inclusion of additional measures of vegetation structure would increase the extent to which edge effects were mediated by vegetation structure in our path model (Appendix S1). To do this, we converted our six vegetation variables into four uncorrelated principal components axes, which together explained 83% of variation in vegetation structure, and used these components as the measures of vegetation structure in our path models. We did not use this approach in our main analysis because the inclusion of principal component axes may have reduced our ability to reject incorrectly specified path models (Appendix S1).

**Figure 1:**
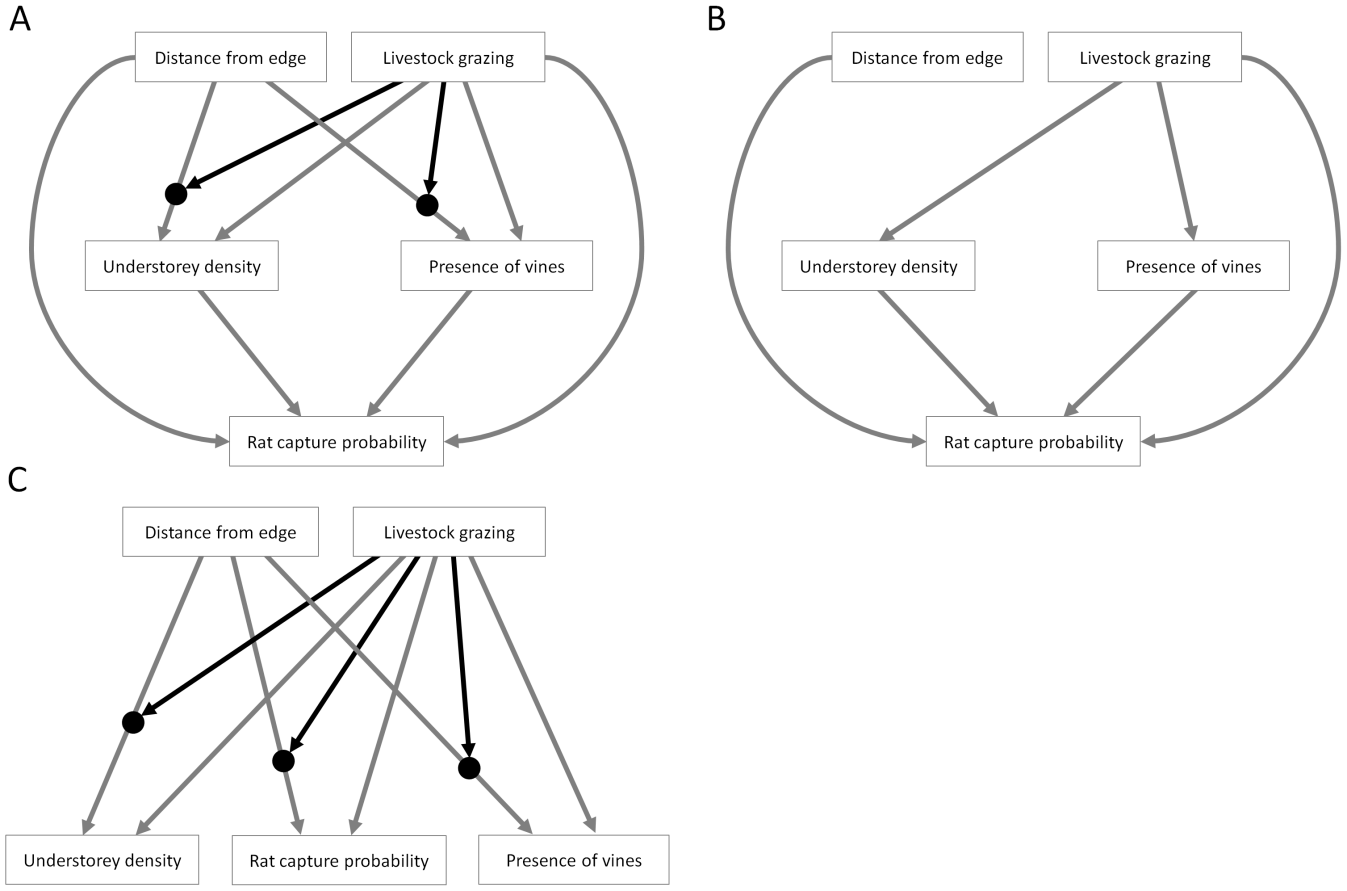
Alternative models of causal relationships between edge distance, grazing, vegetation structure, and rat capture probability. **A**: model hypothesised in this study, in which distance from forest edge and grazing indirectly influenced rat capture probability by driving changes in vegetation structure, and in which grazing altered the strength of the distance from edge effect; **B**: an alternative ‘direct edge effects’ model, where livestock grazing effects on rat capture probability were mediated by vegetation structure, but edge effects were not; **C**: an alternative ‘direct effects only’ model, where distance from forest edge, livestock grazing, and their interaction affected vegetation structure variables and rat capture probability, but where neither edge effects nor livestock grazing effects on rat capture probability were mediated by vegetation structure. Black arrows represent variables which alter the strength of the relationship they feed into (i.e. interaction effects).

In developing the causal structure of our path model, we initially constructed a ‘full’ version of our hypothesised model which included all possible paths consistent with our hypothesis: (1) distance from edge, livestock grazing, and their interaction all influenced the two vegetation variables, and (2) both vegetation variables affected rat capture probability. We also included direct paths from distance from edge to rat capture probability and from livestock grazing to rat capture probability, to allow for any effects of these variables on rat capture probability that did not operate through our measured vegetation variables (Figure 1a).

We attempted to reduce this full model into a more parsimonious one by dividing it into a series of sub-models, one for each endogenous variable (i.e. a variable with paths leading into it) in the path model. Each of these sub-models was a standard mixed effects model, with the endogenous variable as the response variable and all variables directly ‘upstream’ of it as its predictors [49], [50]. These models included patch as a random factor, and were either linear mixed models (‘LMMs’; vegetation variables as response) or binomial GLMMs (rat capture probability as response). LMMs and GLMMs were fitted with the ‘nlme’ package [51] and ‘lme4’ package, respectively, in R. We fitted these models with restricted maximum likelihood (‘REML’), inspected plots of residuals against fitted values and against each predictor, and where necessary dealt with heterogeneity using nlme's ‘varIdent’ function [52]. We then attempted to reduce the number of paths in each sub-model by re-fitting them with maximum likelihood and using backwards selection based on AIC [50].

Reduced models were re-fitted with REML, re-validated, and then reassembled into our final, most parsimonious path model [50]. The overall fit of this final path model was then tested using Shipley's [49] d-sep test for generalised multilevel path models. This test involves identifying a set of ‘independence claims’ (pairs of variables which should be uncorrelated under statistical control if the path model is correct), then measuring whether observed levels of correlation across all independence claims can be explained by random variation [49]. For our final path model, path coefficients were calculated as the estimated slopes of each of the retained variables in the final LMM and GLMM sub-models [49].

Path analyses sometimes calculate path coefficients based on standardised variables to enable measurement of the relative strengths of the different pathways (e.g. [50]). We did not take this approach, instead using unstandardised versions of all variables. This was because different path coefficients had different interpretations as a result of different transformations of predictor variables (distance from edge was square root transformed, while other variables were untransformed; see below) and different link functions for response variables (logit link for rat captures, identity link for other variables) used in models.

#### Alternative causal models

A lack of evidence against a hypothesised path model does not necessarily mean that it is correct, so researchers should also consider alternative models that could plausibly explain relationships among variables [53]. We developed two alternative models that could plausibly explain relationships between distance from edge, livestock grazing, vegetation, and rat capture probability: (1) a ‘direct edge effects’ model, where livestock grazing effects on rat capture probability were mediated by vegetation structure, but edge effects were not (Figure 1b); and (2) a ‘direct effects only’ model, where neither edge effects nor livestock grazing effects on rat capture probability were mediated by vegetation structure (Figure 1c). We used the d-sep test [49] to determine whether there was also support for these causal relationships among variables.

#### Data transformations

Distance from edge was square-root transformed prior to all analyses, for two reasons. First, some of the paths in our models were modelled with LMMs, and the square-root performed best out of a range of transformations at linearising relationships among variables. Second, this transformation forced fitted values of the relationship between distance from edge and rat capture probability to be a decelerating curve, rather than the sigmoidal curve fitted by a standard binomial GLMM on untransformed predictor variables. We considered a decelerating curve to be a more biologically realistic shape for edge effects, because changes are expected to be greatest near the forest edge.

## Results

We caught rats in 22% of our traps across all patches (108 rats across 488 traps), all of which were ship rats. Rat capture rates varied widely among patches, with the proportion of traps that caught rats ranging from 0% to 65%.

### Net effects of distance from edge and livestock grazing on rat capture probability

In our initial ‘net effects’ models of the effects of distance from edge and livestock grazing on rat capture probability, AIC suggested the global model was best (i.e an effect of distance from edge, grazing, and their interaction), but there was a similar weight of evidence (Δ AIC <1) for all three models that included a distance effect (i.e. the global model, the distance and grazing main effects model, and the distance-only model). In contrast, there was little evidence to support the livestock grazing-only or intercept-only models (Δ AIC >4) (Table S2). We therefore used a model averaging approach to estimate parameters across the three models that included a distance effect.

**Figure 2:**
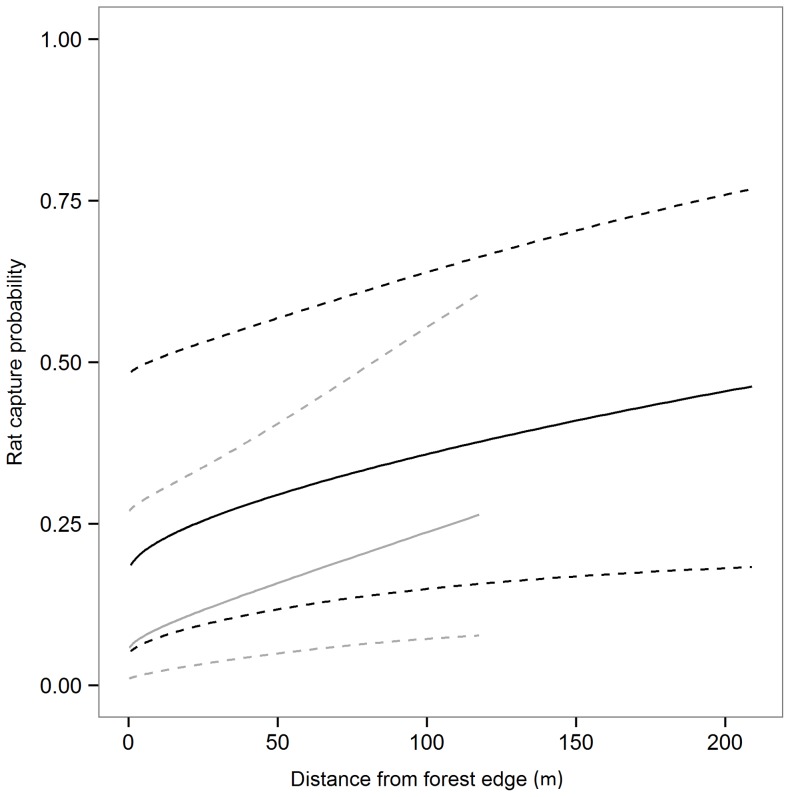
Model-predicted values for the relationship between rat capture probability and distance from forest edge. Values were predicted separately for those patches that were grazed by livestock (grey lines) and those that were not (black lines). Dotted lines show 95% confidence intervals for predicted values. The truncated values for grazed patches reflect the reduced range over which edge distances were measured in these patches.

Fitted values from these model-averaged parameters showed that rat capture probability clearly increased with distance from edge. In forest fragments grazed by livestock, capture probabilities were estimated to be 78% lower at the forest edge than 118 m into the forest interior (the greatest distance measured in unfenced fragments) (Figure 2). This trend was similar in fragments from which livestock were excluded, with capture probabilities estimated to be 51% lower at the forest edge than 118 m into the forest interior. These ungrazed fragments also tended to have higher rat capture probabilities for a given distance from the forest edge (Figure 2). However, unconditional confidence intervals marginally overlapped zero for the effects of distance, grazing, and their interaction (Table S3). Nonetheless, we believe there is strong support for an effect of edge distance on rat capture rates. First, unconditional confidence intervals are conservative in that they incorporate uncertainty in model structure. Second, only those models which included a distance effect were well-supported as judged by AIC (Table S2), and a distance main effect was significant in each of these models (distance-only model: p = 0.01; main effects model: p = 0.02; full model: p = 0.01).

### Mediating effects of vegetation structure on edge effects and livestock grazing effects

**Figure 3:**
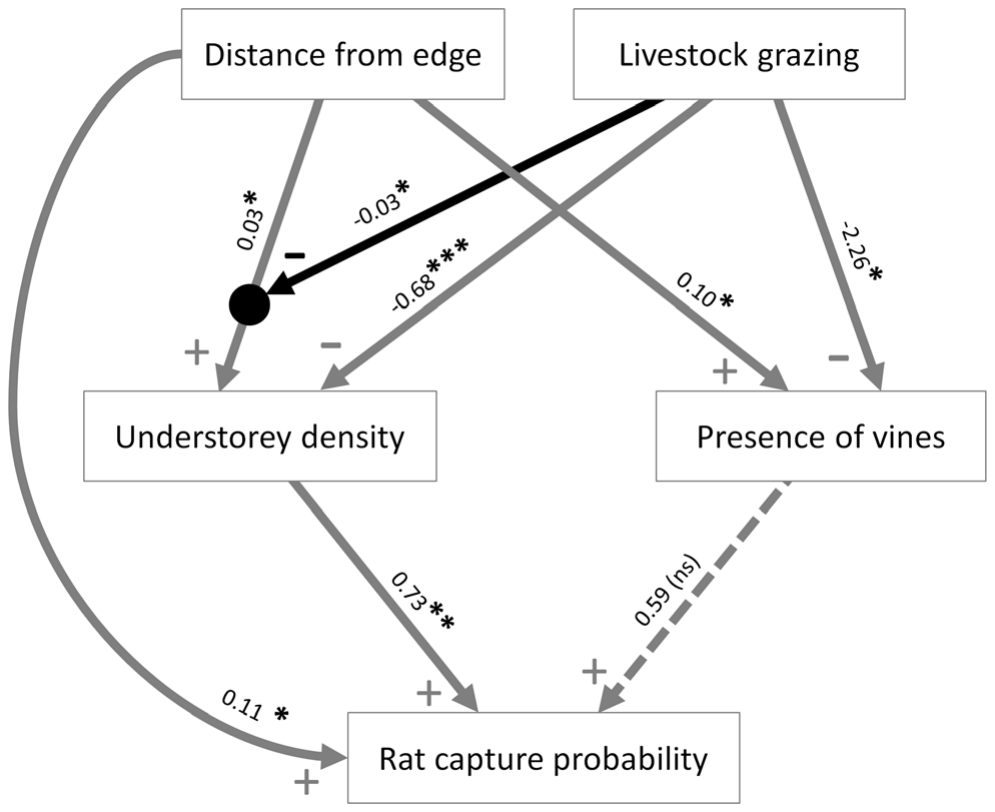
Final path model showing relationships between distance from forest edge, grazing, vegetation structure, and rat capture probability. Plus/minus symbols at each arrow head show a positive/negative effect, and asterisks denote significance of each path: ***<0.001; **<0.01; *<0.05; ns  =  not significant. Dotted arrows show non-significant paths, and black arrows show variables which alter the strength of the relationship they feed into (i.e. interaction effects). Note that path coefficients are not directly comparable because of different scales of measurement of predictor variables (distance square root transformed; other variables untransformed) and different link functions for response variables (logit link for rat capture probability; identity link for other variables) used in models.

Backwards variable selection within sub-models allowed us to simplify our hypothesised path model (i.e. the model in which the effects of forest edges and livestock grazing on rat capture probability were mediated by vegetation structure; Figure 1a). This resulted in the removal of the direct effect of livestock grazing on rat capture probability, as well as the livestock grazing by distance from edge interaction on the presence of vines (Figure 3). This model provided a good fit to the data (d-sep test, χ^2^ = 8.98, df = 8, p = 0.344). In contrast, our two alternative path models which also could have plausibly explained relationships among distance from edge, livestock grazing, vegetation structure and rat capture probability (Figure 1b,c) were both rejected by the d-sep test (p<0.05), providing further support for our hypothesised path model.

In line with our causal hypothesis, path coefficients for the final reduced path model (Figure 3) suggested that the effects of distance from edge on rat capture probability were largely mediated by changes in vegetation structure: the understorey became denser and vines became more common with increasing distance from the forest edge, and these in turn were associated with increased rat capture probability. The model also suggested a significant direct effect of distance from edge on rat capture probability which operated independently of our measures of vegetation structure. However, part of this direct effect of distance from edge may have included vegetation-mediated effects which were not captured by our two vegetation structure variables. Indeed, a sensitivity test found that this direct effect weakened and became non-significant when the model included additional measured variation in vegetation structure (Figure S1). Our path model also suggested that livestock grazing had a negative effect on rat capture probability, which was mediated by changes in vegetation structure (Figure 3). Finally, the model suggested that grazing altered the strength of the vegetation-mediated effect of distance from edge on rat capture probability, with the effects of distance from edge on understorey density being weaker in grazed than ungrazed fragments (Figure 3).

## Discussion

Predicting and managing the effects of forest edges on nest predation rates requires an understanding of the mechanisms driving their occurrence, but few studies have moved beyond describing spatial variation in nest predation rates to testing hypotheses about the underlying causes [5], [6], [18], [54]. In this study, path analysis allowed us to identify edge-driven changes in vegetation structure as a major driver of edge effects on the capture rates of ship rats, a globally invasive nest predator. Vegetation structure has been found to correlate with nest predation rates previously [55]–[57], although we know of no studies that have identified change in vegetation structure as a driver of edge effects on nest predation. We also found that the relationships between forest edges, vegetation structure, and ship rats gave rise to ‘reverse’ edge effects in our study system, in which rat capture rates were higher in the interior of forest fragments than at the forest edge. This finding contrasts with the common assumption that forest edges increase nest predation rates [9], and supports a growing body of evidence which suggests that edge effects on nest predation rates are inconsistent, context dependent, and ultimately unpredictable without an understanding of the mechanisms involved (e.g. [13], [15], [16], [21], [56], [58]).

The ‘within patch’ mechanism underpinning edge responses in our study system contrasts with the widely held belief that edge effects on nest predation rates result from large-scale processes that operate beyond individual forest patches (e.g. [6], [13], [18], [58]). In particular, the most frequently cited mechanism for edge effects on nest predation rates is ‘matrix spill-over’, where generalist predators reach high densities in adjacent matrix land uses and then forage in forest remnants, leading to elevated nest predation at the forest edge [6], [13], [20], [59], [60]. Matrix spill-over is likely to cause edge effects wherever dominant nest predators (1) increase in response to matrix land uses and (2) range across both forest and matrix habitats. However, while this has often proven true for predator communities in the temperate northern hemisphere studies that have dominated the literature [16], [18] it was unlikely to be the case in our study system, because ship rats typically decrease in response to pastoral land use and remain within forest habitats [31], [61].

Instead, ship rats responded to edge-driven changes in habitat within forest patches, resulting in capture rates that were higher in the forest interior than at the forest edge. This response may have occurred via the movement of individuals, since the typical home range length of a ship rat (100–300 m; [35]) would encompass multiple traps, or via population-level changes in local abundance. It may also underlie the results of a previous study which found the same ‘reverse’ edge effects on ship rat capture rates in New Zealand forests [33]. We expect low matrix spill-over for other matrix types in New Zealand (for example, urban areas, plantation forests, or cropland), since ship rats also decrease in response to these land uses [31], [62], [63].

We suggest that where dominant nest predators are primarily forest species that do not range across forest and matrix habitats, edge effects on nest predation rates may be more closely tied to changes in habitat within forest patches than to changes in habitat in the wider landscape. In these cases, forest edges may have positive, neutral, or negative effects on nest predation rates, depending on if and how important resources for dominant predators vary with distance from the forest edge. This is in line with suggestions that variability in edge effects on nest predation may largely result from differences in habitat use by different predator communities [6], [15], [16], [21].

Our net effects models suggested that livestock grazing reduced the capture rates of ship rats, and path analysis indicated that this effect was also mediated by vegetation structure. These results support previous findings on the effects of livestock grazing on ship rat capture rates in New Zealand [35]. In contrast, evidence for our hypothesis that livestock grazing altered the strength of edge effects on rat capture rates was equivocal. In support of this hypothesis, our path analysis suggested that grazing moderated the effect of distance from edge on vegetation structure, which in turn influenced rat capture probability. Similarly, our AIC-best net effects model specified that the effect of distance from edge on rat capture probability depended on whether fragments were grazed. However, there was similar support for models in which edge effects operated independently of grazing effects, and model-averaged fitted values suggested that any moderating influence of livestock grazing on the effect of forest edges on rat capture rates was weak.

Although we identified vegetation structure as a major mechanism underlying both edge effects and livestock grazing effects on rat capture rates, vegetation structure was potentially correlated with a large number of other variables that determine habitat quality for ship rats. For example, microclimate, vegetation composition, and densities of ship rat prey (primarily invertebrates; [31]) and ship rat predators (primarily stoats *Mustela erminea*, but also feral cats *Felis catus*, weasels *M. nivalis*, and ferrets *M. putorius*); [31]) may have been correlated with vegetation structure (e.g. [5], [64], [65]) and were likely to influence habitat quality for ship rats. Moreover, while our path model included understorey density and presence of vines as our measures of vegetation structure, these variables were also correlated with our additional vegetation variables (canopy height, canopy cover, cover of dead tree fern fronds, and cover of living vegetation <1.5 m above ground). As such, ship rats may not have been responding to understorey density and the presence of vines per se, but these variables may instead have been proxies for the true measures of habitat quality that influenced ship rat capture rates.

It is also important to point out that the density of birds' nests might well be correlated with vegetation structure. If so, it is possible that increases in rat density could be balanced by increases in nest density, such that ship rat capture rates become decoupled from per capita (i.e. per-nest) predation rates. Such a decoupling could occur, for example, if rats become satiated at high nest densities and decrease their per capita rate of predation (i.e. a Type II or Type III functional response; [66]). We know of no studies which have found that nest density increases with increasing vegetation structure for any of the native bird species found in our study area, although this relationship has been found for a species which occurs elsewhere in New Zealand (saddleback *Philesturnus carunculatus*; [67]).

Our path analysis suggested that there were additional effects of distance from edge on rat capture probability that were not mediated by understorey density or presence of vines. While this may reflect the fact that forest edges affect rat capture rates in ways unrelated to vegetation structure, it is also likely that this ‘direct path’ at least partially represented the effects of vegetation structure on rat capture rates that were not captured by understorey density and vines alone. Indeed, in a sensitivity analysis that included all six of our original vegetation variables as an orthogonal set of principle components (to avoid potential reciprocal effects among variables), the direct path between distance from edge and rat capture probability weakened and became non-significant (Figure S1). This suggests that the observed direct path between distance from edge and rat capture probability may be a consequence of the small number of variables we used to characterise vegetation structure.

Our study highlights the value of a mechanistic approach for understanding variability in edge effects on nest predation rates and for predicting how forest edges will affect the abundance or impacts of ship rats elsewhere. Our approach suggested that changes in vegetation structure largely mediated both the occurrence of edge effects on rat capture probability and variability in their strength due to livestock grazing. This implies that edge effects on ship rats can be predicted from the response of vegetation structure to forest edges. Although our measures of vegetation density increased with distance from the forest edge, the reverse is often true [5], [68], even within other lowland podocarp-broadleaf forests in New Zealand [64]. This suggests that edge effects on ship rats may reverse in other systems. Support for this suggestion comes from a previous study of edge effects on ship rats in the Canary Islands, where vegetation density was higher at the edge than in the forest interior [69]. As predicted by our path model, ship rat density was also higher at the forest edge, the opposite trend to that found in our study [69]. These results suggest that variability in edge effects on ship rats can be understood and predicted, even to the point of changing sign, based on an understanding of how vegetation structure responds to forest edges. Our results also raise the possibility of reducing the impacts of ship rats by reducing the structural complexity of vegetation within patches, although this strategy would generally be inappropriate in New Zealand given that native forest of high conservation value is often characterised by a structurally complex understorey [35].

Path analysis is a promising approach for understanding the roles of forest edges and local habitat in influencing nest predation rates. However, studies of edge effects by their very nature tend to produce hierarchically structured data (i.e. measurements are taken at multiple distances from edge across several forest patches) and non-normal response variables (e.g. predator capture probability, predator counts, or daily predation rates of real or artificial nests), making analysis with traditional path modelling methods difficult [49]. Recent methodological developments that enable the inclusion of random effects and non-normal response variables in path models, such as generalised multilevel confirmatory path analysis, make path analysis a powerful approach for understanding the mechanisms that underlie edge-driven changes in nest predation rates.

## Supporting Information

Figure S1 **Final path model showing estimated causal relationships between distance from the forest edge, livestock grazing, vegetation structure (principal components Veg PC1-4) and probability of rat capture.** Prior to path analysis vegetation variables were converted to six principal components, but only the first four were related to distance from edge, livestock grazing, or rat capture probability, so Veg PC5–6 were omitted from the analysis. The first four components explained 34%, 22%, 15%, and 12% of the total measured variance in vegetation structure respectively. Plus/minus symbols at each arrow head show a positive/negative effect, and asterisks denote significance of each path: ***<0.001; **<0.01; *<0.05; ns  =  not significant. Dotted arrows show non-significant paths, and black arrows show variables which alter the strength of the relationship they feed into (i.e. interaction effects). Note that path coefficients are not directly comparable because of different scales of measurement of predictor variables (distance square root transformed; other variables untransformed) and different link functions for response variables (logit link for rat capture probability; identity link for other variables) used in models.
(TIF)

Table S1 **Summary statistics for patch size and sampling effort (number of rat traps) per patch for grazed and ungrazed patches.**
(DOCX)

Table S2 **Levels of support for candidate models used to test the effects of distance from forest edge, livestock grazing, and their interaction on rat capture probability.** ΔAIC measures change in AIC relative to the best model, while the Akaike weight *w_i_* gives the probability that model *i* is the most parsimonious in the set. The table shows fixed terms only, but candidate models also included a patch-level random intercept to model non-independence of rat traps within the same forest patch.
(DOCX)

Table S3 **Parameter estimates and 95% confidence intervals (in parentheses) for effects of distance from edge, livestock grazing, and their interaction on rat capture probability.** Multiple candidate models were supported by the data (ΔAIC <4; see Table S1), so parameter estimates and confidence intervals were averaged across these models to account for uncertainty in model structure. Parameter estimates are given on the scale of the logit link used in binomial GLMMs.
(DOCX)

Table S4 **Data used in the analyses.**
(XLSX)

Appendix S1 **Reanalysis of path model using all vegetation variables.**
(DOCX)
