# Supplementary material for: Discriminating the Drivers of Edge Effects on Nest Predation: Forest Edges Reduce Capture Rates of Ship Rats (Rattus rattus), a Globally Invasive Nest Predator, by Altering Vegetation Structure
Source: PLoS One. 2014 Nov 20;9(11):e113098. doi: 10.1371/journal.pone.0113098 (PMC4239037; doi:10.1371/journal.pone.0113098)
Supplement: Table S1 — Summary statistics for patch size and sampling effort (number of rat traps) per patch for grazed and ungrazed patches. (DOCX) [file pone.0113098.s002.docx]

**Table S1.** Summary statistics for patch size and sampling effort (number of rat traps) per patch for grazed and ungrazed patches.

| **Variable** | **Grazed patches** | | | |  | **Ungrazed patches** | | | |  |
| --- | --- | --- | --- | --- | --- | --- | --- | --- | --- | --- |
|  | n* | Min | Mean | Max |  | n* | Min | Mean | Max |  |
|  |  |  |  |  |  |  |  |  |  |  |
| Patch size (ha) | 7 | 8.2 | 12.0 | 16.0 |  | 8 | 1.8 | 10.2 | 18.9 |  |
| Sampling effort | 7 | 5 | 30.2 | 54 |  | 8 | 6 | 34.5 | 76 |  |
|  |  |  |  |  |  |  |  |  |  |  |

*Includes two patches which contained both grazed and ungrazed sections
