# Supplementary material for: Discriminating the Drivers of Edge Effects on Nest Predation: Forest Edges Reduce Capture Rates of Ship Rats (Rattus rattus), a Globally Invasive Nest Predator, by Altering Vegetation Structure
Source: PLoS One. 2014 Nov 20;9(11):e113098. doi: 10.1371/journal.pone.0113098 (PMC4239037; doi:10.1371/journal.pone.0113098)
Supplement: Table S2 — Levels of support for candidate models used to test the effects of distance from forest edge, livestock grazing, and their interaction on rat capture probability. ΔAIC measures change in AIC relative to the best model, while the Akaike weight wi gives the probability that model i is the most parsimonious in the set. The table shows fixed terms only, but candidate models also included a patch-level random intercept to model non-independence of rat traps within the same forest patch. (DOCX) [file pone.0113098.s003.docx]

**Table S2.** Levels of support for candidate models used to test the effects of distance from forest edge, livestock grazing, and their interaction on rat capture probability. ΔAIC measures change in AIC relative to the best model, while the Akaike weight *w_i_* gives the probability that model *i* is the most parsimonious in the set. The table shows fixed terms only, but candidate models also included a patch-level random intercept to model non-independence of rat traps within the same forest patch.

| **Model** | | **ΔAIC** | ***w_i_*** |  |
| --- | --- | --- | --- | --- |
|  | |  |  |  |
| Distance + Grazing + Distance:Grazing | | 0.00 | 0. 38 |  |
| Distance | | 0.62 | 0. 28 |  |
| Distance + Grazing | | 0.64 | 0. 27 |  |
| Grazing | | 4.26 | 0.05 |  |
| 1* | | 4.95 | 0.03 |  |
|  |  | | | |

*Null model containing intercept only
