# Supplementary material for: Discriminating the Drivers of Edge Effects on Nest Predation: Forest Edges Reduce Capture Rates of Ship Rats (Rattus rattus), a Globally Invasive Nest Predator, by Altering Vegetation Structure
Source: PLoS One. 2014 Nov 20;9(11):e113098. doi: 10.1371/journal.pone.0113098 (PMC4239037; doi:10.1371/journal.pone.0113098)
Supplement: Table S3 — Parameter estimates and 95% confidence intervals (in parentheses) for effects of distance from edge, livestock grazing, and their interaction on rat capture probability. Multiple candidate models were supported by the data (ΔAIC <4; see Table S1), so parameter estimates and confidence intervals were averaged across these models to account for uncertainty in model structure. Parameter estimates are given on the scale of the logit link used in binomial GLMMs. (DOCX) [file pone.0113098.s004.docx]

**Table S3.** Parameter estimates and 95% confidence intervals (in parentheses) for effects of distance from edge, livestock grazing, and their interaction on rat capture probability. Multiple candidate models were supported by the data (ΔAIC<4; see Table S1), so parameter estimates and confidence intervals were averaged across these models to account for uncertainty in model structure. Parameter estimates are given on the scale of the logit link used in binomial GLMMs.

| **Model term** | **Estimate** |
| --- | --- |
|  |  |
| Intercept^ǂ^ | -1.17 (-3.03, -0.08) |
| Distance | 0.10 (-0.02, 0.21) |
| Grazing | -1.90 (-4.17, 0.38) |
| Distance:Grazing interaction | 0.18 (-0.04, 0.40) |
|  |  |
