# Supplementary material for: Discriminating the Drivers of Edge Effects on Nest Predation: Forest Edges Reduce Capture Rates of Ship Rats (Rattus rattus), a Globally Invasive Nest Predator, by Altering Vegetation Structure
Source: PLoS One. 2014 Nov 20;9(11):e113098. doi: 10.1371/journal.pone.0113098 (PMC4239037; doi:10.1371/journal.pone.0113098)
Supplement: Appendix S1 — Reanalysis of path model using all vegetation variables. (DOCX) [file pone.0113098.s006.docx]

## Appendix S1: reanalysis of path model using all vegetation variables

In our main path analysis, we included only two uncorrelated measures of vegetation structure: understorey density and presence of vines. This was because there may have been reciprocal effects among our other measures of vegetation structure, which cannot be handled by generalised multilevel confirmatory path analysis [1].

However, because these two vegetation variables reflected only part of the total variation in vegetation structure, any significant effects of vegetation structure on rat capture probability not captured by these two variables would appear in the path analysis as a 'direct effect' of forest edges on rat capture probability. Our main path analysis did indeed suggest that there was a direct effect of forest edges on rat capture probability that did not operate through understorey density or presence of vines, and we were interested in whether this was due to additional variation in vegetation structure that was not captured by these two variables. To examine this, we reanalysed our path model including all six of our original vegetation variables after converting them to orthogonal axes of a Principal Components Analysis (PCA) ordination (hereafter 'components') which were uncorrelated and thus could not have strong reciprocal effects on one another.

While PCA allowed us to include all measured variation in vegetation structure, we consider this approach inferior to the method used in our main analysis (i.e. removing correlated vegetation variables prior to path analysis). This is because the inclusion of independence claims between components was likely to reduce the ability of the d-sep test to reject unsuitable models. This test measures whether overall levels of correlation across all independence claims (i.e. pairs of variables which should be uncorrelated under statistical control if the path model is correct) can be explained by random variation. However, principal components are less correlated with each other than would be expected by chance, and this may have compensated for unacceptably high levels of correlation among other independence claims.

In conducting this analysis, we recognise that standard PCA does not account for nested data structures. As a result, although a standard PCA on vegetation variables would create components that were uncorrelated across the dataset as a whole, it might still create components that were correlated within individual patches. To counter this problem, we used the ‘phyl.pca’ function in the R ‘phytools’ package [2] to conduct a hierarchically structured phylogenetic PCA. This function was developed as a means to analyse species traits while accounting for non-independence among species due to shared ancestry [3], an analogous situation to non-independence among observations due to nesting within patches. The ‘phyl.pca’ function required as input a phylogeny to specify how our observations were 'related' to each other. To create this, we specified that all observations from the same patch shared a common ancestor (i.e. shared a common forest patch; representing the patch-level average for each variable), with all forest patches being descended from a single common ancestor (representing the population-level average for each variable). All branch lengths were arbitrarily set at 1, but results of the PCA were identical for other branch lengths as long as these were constant within each level. The hierarchically-structured PCA produced components which captured the full range of variation in our measured vegetation variables and which were uncorrelated both across the dataset as a whole and within patches. We based our phylogenetic PCA on the correlation (c.f. covariance) matrix of vegetation variables since variables were measured on very different scales. We used an approach identical to that described in our main analysis to reduce the path model to a more parsimonious one, test the adequacy of model structure, and calculate path coefficients for the reduced model (see main text).

Variable reduction in sub-models allowed us to simplify our full model considerably, and this final model predictably had a high level of support (d-sep test, χ^2^ = 25.87, df = 32, p= 0.769). However, the model was also well supported when the independence claims among principal components were omitted (χ^2^ = 21.94, df = 20, p = 0. 344). The final path model (Figure S1), based on all measured variability in vegetation structure, gave qualitatively identical results to our main analysis which used only understorey density and the presence of vines as measures of vegetation structure: (1) the model structure was well supported (noting that our ability to reject model structure was compromised by the inclusion of principal components); (2) this model suggested that major effects of distance from edge, livestock grazing, and their interaction on rat capture probability were mediated by changes in vegetation structure; and (3) there were no direct effects of livestock grazing, or a livestock grazing by distance from edge interaction, on rat capture probability. In particular, the inclusion of this additional measured variation in vegetation structure weakened the estimated direct effect of distance from edge on rat capture probability. Compared with the path model in the main analysis, the estimated slope of the relationship decreased (from 0.11 to 0.08) and became non-significant (p = 0.13). Moreover, while the best-fit model included this direct path, the fit of the model which excluded it was essentially equivalent (Δ AIC = 0.2). Together, these results suggest that the significant direct effect of forest edges on rat capture probability found in our main analysis may represent vegetation-mediated effects of edges on rat capture probability which operate through aspects of vegetation structure not captured by understorey density or presence of vines.

## References

1. Shipley B (2009) Confirmatory path analysis in a generalized multilevel context. Ecology 90: 363-368.

2. Revell L (2014) phytools: Phylogenetic tools for comparative biology. Version 0.3-93.

3. Revell L (2009) Size-correction and principal components for interspecific comparative studies. Evolution 63: 3258-3268.
